# Prognostic significance of immunoscore related markers in bladder cancer

**DOI:** 10.1186/s12894-022-01085-6

**Published:** 2022-08-29

**Authors:** Ali Ariafar, Alireza Sanati, Simin Ahmadvand, Golsa Shekarkhar, Akbar Safaei, Zahra Shayan, Zahra Faghih

**Affiliations:** 1 Department of Urology, School of Medicine, Shiraz University of Medical Sciences, Shiraz, Iran; 2 Shiraz Institute for Cancer Research, School of Medicine, Shiraz University of Medical Sciences, P.O. Box: 71345-1798, Shiraz, Iran; 3 Department of Pathology, School of Medicine, Shiraz University of Medical Sciences, Shiraz, Iran; 4 Department of Biostatistics, Trauma Research Center, School of Medicine, Shiraz University of Medical Sciences, Shiraz, Iran

**Keywords:** Bladder cancer, TILs, CD3, CD8, CD45RO, FOXP3

## Abstract

**Background:**

The significance of total and specific subpopulations of tumor-infiltrating lymphocytes (TILs) in cancer is now well-documented. In the present study, we investigated the relevance of CD3+, CD8 +, CD45RO +, and FOXP3 + TILs to the prognosis and survival of patients with bladder cancer and the disease's clinical-pathological parameters.

**Methods:**

Infiltration of each subset was immunohistochemically evaluated in both stromal and intratumoral regions of tumor tissues from 85 patients with urothelial cell carcinoma of the bladder, with known survival.

**Results:**

Our results indicated that intratumoral CD45RO+ lymphocytes were significantly higher in high-grade tumors than in low-grade ones (P = 0.028). The frequencies of intratumoral CD3+ (P = 0.002), CD8 + (P = 0.008), intratumoral (P = 0.002), and stromal (P = 0.017) CD45RO+ lymphocytes were also higher in patients with muscular invasion than those without invasion. The frequencies of intratumoral CD3+ (P = 0.043), CD8+ (P = 0.003), CD45RO+ (P = 0.023), and total CD45RO+ (P = 0.015), showed variation in patients with different T-stage, as well; mostly increased in T2 versus Ta and T1. Comparing patients in different stages revealed an increase in the frequencies of total CD3+ (P = 0.011), intratumoral CD3+ (P = 0.006), total CD8+ (P = 0.012), intratumoral CD8+ (P = 0.009) and stromal CD8+ (P = 0.034), as well as total and stromal CD45RO+ lymphocytes (P = 0.01 and P = 0.034, respectively) in stage II comparing to stage I, while the frequencies of stromal CD3+ (P = 0.077) and CD8+ (P = 0.053) cells tended to be decreased in stage III compared to stage II.

**Conclusions:**

We collectively observed that the frequency of immune cells, especially CD45RO+, CD3+, and CD8+ lymphocytes, were significantly higher in early-progressed tumors. This observation could be explained by continuous and prolonged stimulation of immune cells with tumor antigens during tumor progression or an increase in the recruiting factors, especially in the early stages, to eliminate tumor cells. However, with tumor progression to the late stages, the inhibitory microenvironment provided by tumor cells suppresses or changes the functionality of the effector and memory immune cells to help tumor growth. However, more functional studies with larger sample sizes are needed to reveal the real status of the immune system in patients with bladder cancer.

## Background

Prediction of outcome and prognosis of patients with bladder cancer (BC), like many other solid tumors, is mainly based on the histopathological evaluation of resected primary tumor, including local tumor invasion (T), lymph node involvement (N), and metastasis (M). These data collectively assess pathological disease stage—TNM classification system—which is currently a vital parameter to estimate disease prognosis and response to treatment [1]. However, it is now well-documented that this traditional classification system is not a good predictor of prognosis [2]. One of the failure reasons for this system seems to be the lack of attention to the impact of the tumor microenvironment, especially the host immune system, on tumor progression [3].

The presence of immune cells within the tumor microenvironment has long been proved histologically. Many reports support the hypothesis that the host immune system strictly controls cancer development. Accordingly, an immune-based classification system, also known as Immunoscore, has been recently introduced in cancer, surpassing the conventional TNM-stage in predicting patients' disease-free survival (DFS) and overall survival (OS) [4]. However, the prognostic significance of tumor-infiltrating lymphocytes (TILs) in BC has been rarely investigated, mainly focused on limited subsets in invasive BC [5–8]. Thus, in the present study, we investigated the prognostic relevance of Immunoscore related markers, including CD3, CD8, CD45RO (effector/memory), and FOXP3 (inhibitory), in both superficial and invasive BC.

## Materials and methods

### Patients

The study population was selected among 300 patients with BC subjected to transurethral resection of bladder tumor (TURB) or radical cystectomy between 2011 and 2014 in the hospitals affiliated with Shiraz University of Medical Sciences, Iran. The patients received no preoperative BCG, chemo- or radio-therapy before or during surgery. Since the frequency of subtypes other than urothelial carcinoma (UC) was extremely low (less than 5%), to have a uniform population, only patients with urothelial carcinoma of the bladder were selected for further evaluation. Survival status, date of the last follow-up, and for dead patients, date of death were obtained from their records. Patients with incomplete data were removed, and finally, 85 patients were selected for immunohistochemistry staining and analysis. The mean age of patients at the time of diagnosis was 66.96 ± 11.65 years (40–87 years). The mean follow-up period was 30.9 months (1 to 67.5 months). During this time, 40 patients (47.1%) died; however, whether their death was due to cancer or age-related remained clarified. The clinicopathological characteristics of the patients are detailed in Table 1.

**Table 1:** Clinicopathological characteristics of patients with bladder cancer

Characteristics		N (%)	
Age (years)			**Mean (min–max):** 66.96 ± 11.65 (40–87) **Median:** 68.00
Survival	Alive	45 (52.9%)	
	Dead	40 (47.1%)	
Overall survival (month)			**Mean (min–max):** 30.9 ± 19.98(1.1–67.73) **Median**: 24.07
T-stage	Ta	4 (4.7%)	
	T1	27 (31.8%)	
	T2	33 (38.8%)	
	T3	9 (10.6%)	
	T4	12 (14.1%)	
TNM-stage	0	5 (5.9%)	
	1	26 (30.6%)	
	2	28 (32.9%)	
	3	26 (30.6%)	
	4	0 (0%)	
Histological grade	Low	27 (32.5%)	
	High	56 (67.5%)	
Lymphovascular invasion	Positive	18 (21.2%)	
	Negative	63 (74.1%)	
	Unreported	4 (4.7%)	
Lymph node status	Not involved	36 (75.0%)	
	Involved	12 (25.0%)	
	Unreported*	37	
Perineural invasion	Positive	28 (32.9%)	
	Negative	55 (64.7%)	
	Unreported	2 (2.4%)	
Muscle invasion	Positive	54 (63.5%)	
	Negative	31 (36.5%)	

*Unreported cases are those who were subjected to TURB

### Immunohistochemistry (IHC)

Hematoxylin and eosin (H and E)-stained slides were reviewed by experienced pathologists to select appropriate blocks having both stromal and intratumoral regions simultaneously. Sections with 3 µm thickness were then prepared and fixed on poly L-Lysine coated slides. Tissue sections were deparaffinized and rehydrated. Following antigen retrieval and blocking by 10% goat serum, the slides were subjected to immunohistochemical staining, using antibodies specific for CD3 (RTU, Diagnostic Biosystems, USA), CD8 (RTU, Diagnostic Biosystems), CD45RO (1/200 dilution, Dako, Denmark,) and FOXP3 (1/100 dilution, Abcam, USA), as well as corresponding isotype-matched antibodies (all from Dako). 10% hydrogen peroxide (H2O2) was also used to quench endogenous peroxidase activity. Visualization was performed using Master Polymer Plus Detection System, (Peroxidase) (Master Diagnostika, Spain) according to manufacturer instruction. The slides were then washed and counterstained with Hematoxylin. To minimize non-specific staining, the best conditions for antigen retrieval and the optimal dilution of each primary antibody were first determined on human tonsil (an enriched tissue for immune cells) as previously described [9].

After IHC-staining, areas from both stromal and intratumoral regions with predominant positively stained lymphocytic infiltrations were chosen by a pathologist blind to the clinical characteristics and outcomes of the patients. Stromal TILs were defined as lymphocytes far from tumor cells and located in the stromal area. Intratumoral TILS were considered the cells in the tumor nets in direct contact with tumor cells. The images were tried to be taken from the same areas for all markers. Fiji image analysis workstation was used to quantify the number of positively stained cells per area unit (square millimeters).

### Statistical analysis

The number of positive cells in each area was separately counted and adjusted to the tumor's area unit (mm2). Count combinations of stained cells in stromal (ST) and intratumoral (IT) regions were considered the total number of cells. Median test was performed to compare the infiltration levels of subpopulations among different groups of patients. OS was determined as the months between diagnosis and death or last follow-up. Cox-regression analysis was used to investigate variables that were associated with OS. Kaplan–Meier curves were applied to compare the survival rate among groups. All analyses were performed using SPSS version 20 (SPSS GmbH Software, Germany), and P values less than 0.05 were considered statistically significant. Statistical graphs were depicted by SPSS and GraphPad Prism 6 (GraphPad Software, San Diego, CA, USA).

## Results

### Immunophenotyping of tumor infiltrating lymphocytes in bladder tumor tissues

**Fig. 1:**
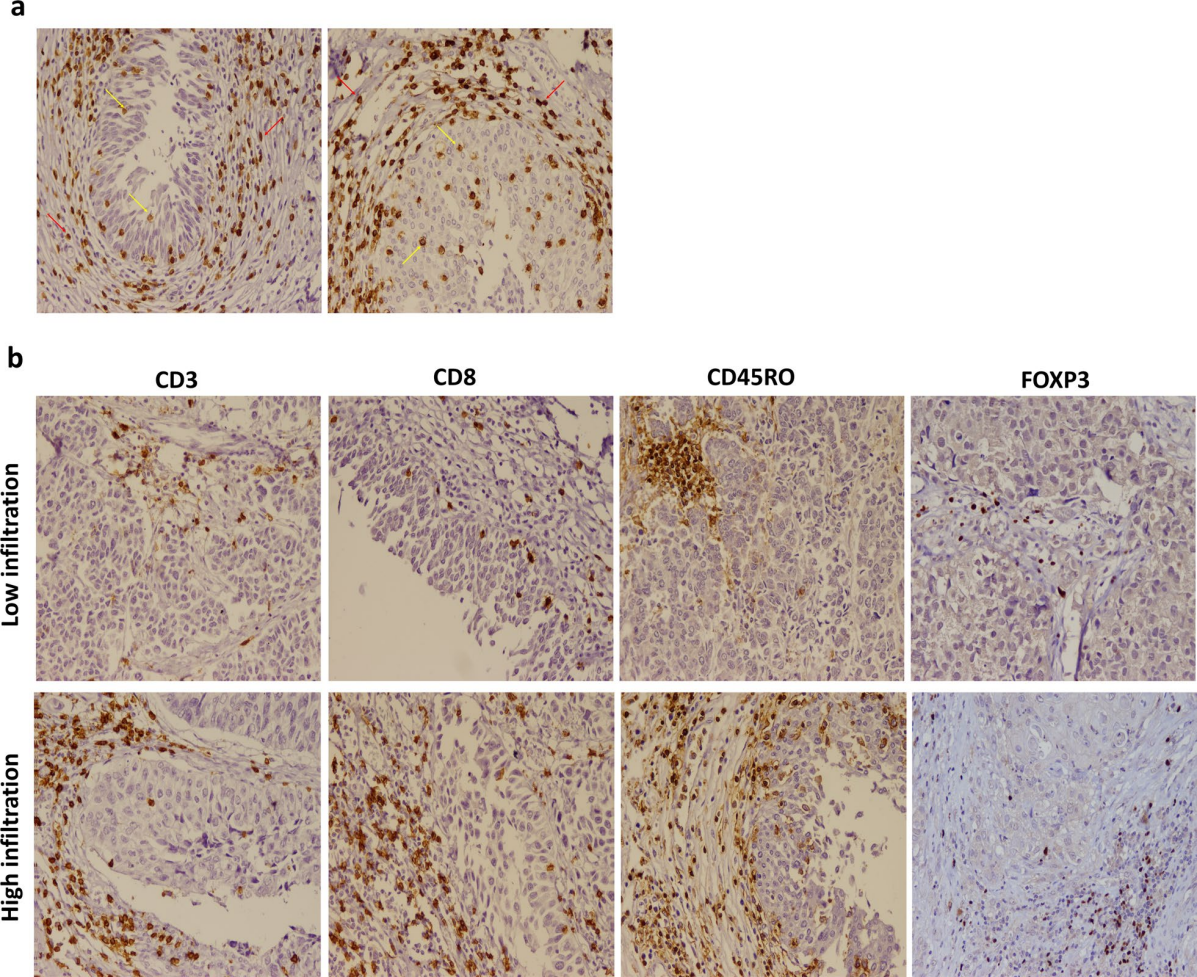
Patterns of lymphocytes’ infiltration in bladder tumor tissues. Infiltration of lymphocytes in both stromal (red arrows) and intratumoral (yellow arrows) was investigated (**a**). Representatives of low (top) and high (bottom) infiltrations of each cell subset are also shown (**b**). The images were captured at 200 × magnification

The presence of lymphocytes expressing CD3, CD8, CD45RO, and FOXP3 markers was histochemically determined in both stromal and intratumoral regions of tumor tissues. The expression pattern in different regions and samples representing low and high infiltration of each subset are illustrated in Fig. 1.

### Mean frequency of immune cells in intratumoral and stromal regions

The number of cells positive for each immune marker was separately determined in stromal and intratumoral regions. As shown in Table 2, the infiltrations of investigated subsets was highly variable; however, they were obviously higher in the stromal regions. CD45RO + cells (77.13 cells/mm2 in ST and 20.58 cells/mm2 in IT), followed by CD3 + cells (74.54 cells/mm2 in ST and 20.05 cells/mm2 in IT), represented the most frequent cells in BC tissue sections, in both regions. On the other hand, FOXP3 + cells with a mean frequency of 28.11 cells/mm2 in the stromal and 4.89 cells/mm2 in the intratumoral region were the least frequent subset. The mean frequency, as well as the minimum and the maximum numbers of investigated subsets, are summarized in Table 2.

**Table 2:** Frequencies of immune cells in stromal and intratumoral regions of bladder tumors

	Intratumoral lymphocytes				Stromal lymphocytes			
	CD3+	CD8+	CD45RO+	FOXP3+	CD3+	CD8+	CD45RO+	FOXP3+
Mean ± SD (cells/mm2)	20.05 ± 33.23	11.75 ± 19.34	20.58 ± 30.96	4.89 ± 9.32	74.54 ± 51.32	30.05 ± 26.72	77.13 ± 57.99	28.11 ± 25.87
Median (IQR)	6 (1–29)	5 (0.5–16)	7 (1–27.50)	1 (0–5)	66 (40.5–102)	25 (12–36.5)	63 (31–103)	24 (8.5–40)
Min–Max	0–179	0–128	0–162	0–59	0–251	0–155	0–291	0–127

### Association of immune cells frequencies and tumor clinicopathological features

**Fig. 2:**
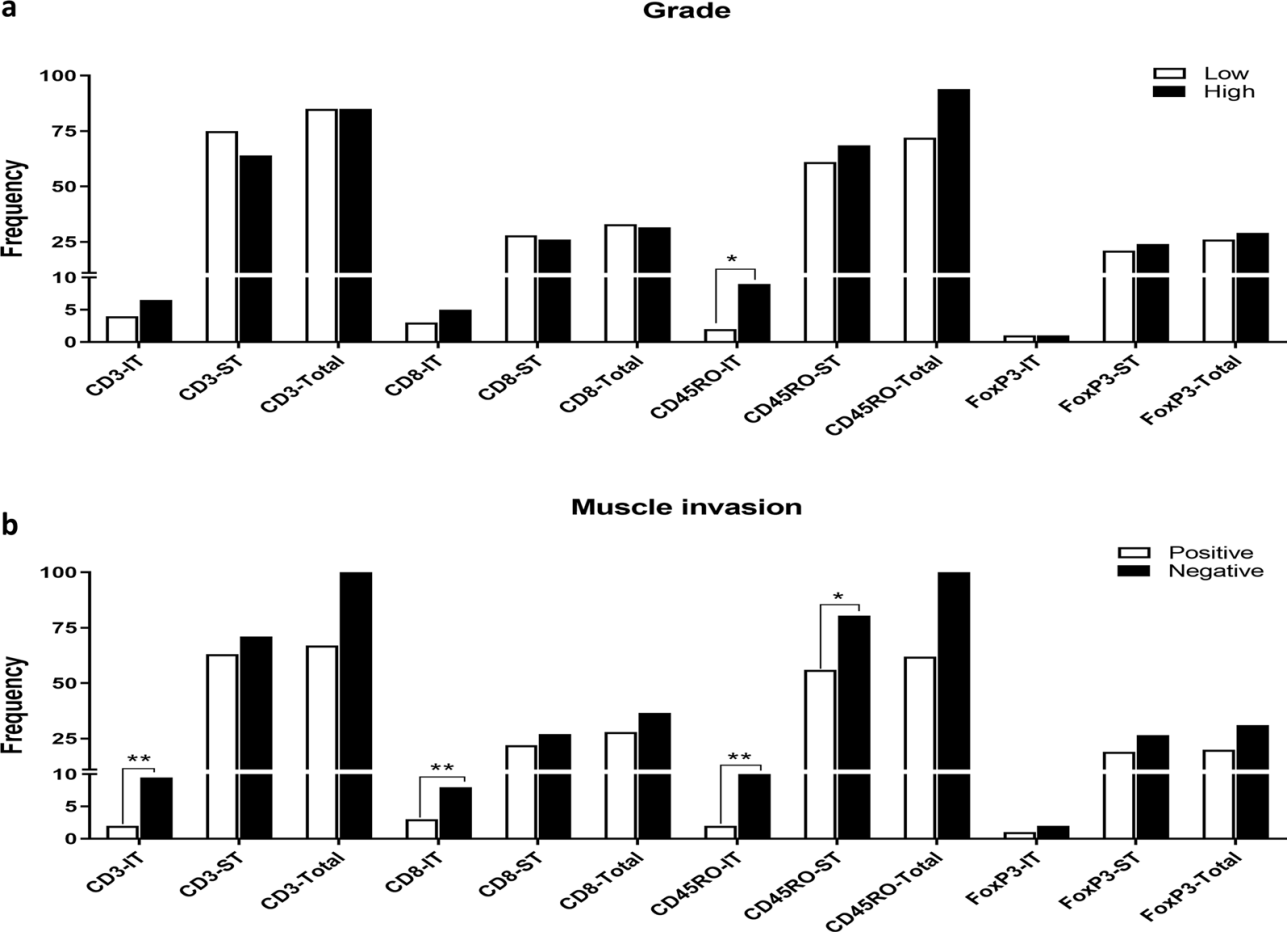
Frequencies of immune cells among clinically relevant parameters of bladder tumors: histological grade (**a**) and muscle invasion (**b**). The data are presented as median. *Difference is significant at 0.05 level (2-tailed), **Difference is significant at 0.01 level (2-tailed). IT: intratumural region, ST: tumor stroma

Following the frequency determination, the association between the prevalence of investigated immune cells in both stromal and intratumoral regions and clinicopathological features of the study population was determined. Comparing the frequencies of different subsets demonstrated that intratumoral CD45RO+ lymphocytes were significantly higher in high-grade tumors than in low-grade ones (P = 0.028, Fig. 2a). The median test also showed that the frequencies of intratumoral CD3+ (P = 0.002), CD8+ (P = 0.008), as well as intratumoral (P = 0.002), and stromal (P = 0.017) CD45RO+ lymphocytes were higher in patients with muscular invasion than those without invasion (Fig. 2b). The frequencies of intratumoral CD3+ (P = 0.043), CD8+ (P = 0.003), CD45RO+ (P = 0.023), FOXP3+ (P = 0.077) cells, and total CD45RO+ (P = 0.015), showed variation in patients with different T-stage, as well; mostly increased in T2 verus Ta and T1.

Comparing patients in different stages revealed an increase in the frequencies of total CD3+ (P = 0.011), intratumoral CD3+ (P = 0.006), total CD8+ (P = 0.012), intratumoral CD8+ (P = 0.009) and stromal CD8+ (P = 0.034), as well as total and stromal CD45RO+ lymphocytes (P = 0.01 and P = 0.034, respectively) in stage II comparing to stage I, while the frequencies of stromal CD3+ (P = 0.077) and CD8+ (P = 0.053) cells tended to be decreased in stage III compared to stage II.

We also investigated the association of different subsets ratios (FOXP3^+^/CD3^+^, FOXP3^+^/CD8^+^, and FOXP3^+^/CD45RO^+^) in different groups of patients. A higher ratio of FOXP3^+^/CD3^+^ was just observed in the tumor tissues of patients with stage III compared to stage I (P = 0.031); however, it did not resist Bonferroni correction. Besides, a higher ratio of stromal FOXP3^+^/CD45RO^+^ was observed in patients without perineural invasion (P = 0.04).

No more significant difference in the frequencies of investigated cells was found in patients with other clinical and pathological parameters, i.e., nodal statues, carcinoma in situ, lymphovascular invasions, and tumor necrosis (P > 0.05).

### Survival analysis

The median follow-up period of patients was 48 months (1–68 months); during this time, 45 patients (52.9%) died. No differences were found between the frequency of immune subpopulations among patients who experienced death, and those who were still alive at their last follow-up, except for the FOXP3+ /CD3+ ratio, which was statistically higher in alive patients [median (IQR): 94.5 (55.50–134.25) vs. 72.00 (46.00–121.50); P = 0.034].

**Fig. 3:**
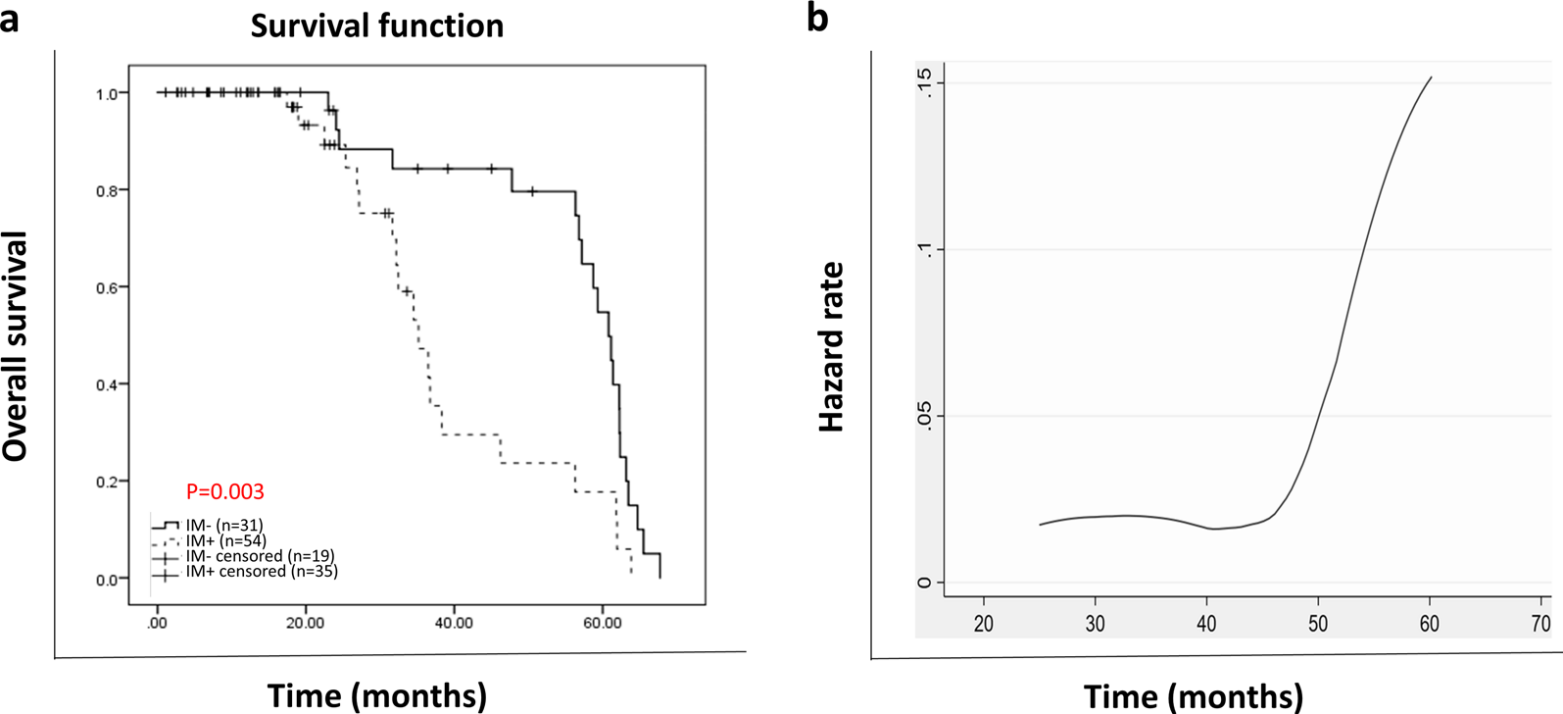
Death Hazard function of patients (follow-up time based on month) and survival plot based on immune cells groups. Patients without invasion showed better survival than those with invasion to the muscular layer (**a**). The hazard rate of death was constant overtime for four years but dramatically increased after that (**b**)

Considering the survival time, the frequency of immune cells showed no significant association. However, the result of the log-rank test indicated that the survival time was significantly longer in the patients without muscle invasion (P = 0.002), lower stages (P = 0.022), and lower T-stage (P = 0.046), as well as those with organ-confined tumors (P = 0.046). We also classified immune cells into two groups with high and low prevalence (based on their medians); however, the difference in the survival time was not significant between the two groups as well. After adjusting by covariates, Cox-regression analysis showed that the muscle invasion was the only significant independent variable that remained in the equation (Hazard ratio = 2.8, P = 0.003) (Fig. 3a). As the hazard function plot in Fig. 3b indicated, the hazard rate of death was constant over four years, and after that, it dramatically increased. While the survival rates continuously decreased from years 1 to 4 (0.93, 0.68, 0.56, and 0.39, respectively).

## Discussion

In the present study, regarding the importance of cytotoxic T (CD8+) and memory (CD45RO+) lymphocytes in eradicating tumor cells and the regulatory role of immune cells expressing FOXP3, we attempted to clarify the role of the immune system based on the frequency of CD3+, CD8+ , CD45RO+ and FOXP3+ lymphocytes in both stromal and intratumoral areas of bladder tumors. Similar to previous studies [10–12], our results revealed a high degree of variation in the prevalence of investigated subsets in different patients. However, in general, CD45RO+ lymphocytes followed by CD3+ cells were the most frequent subsets in both areas of the bladder tumor tissues, while FOXP3+ cells showed the lowest prevalence.

CD45RO is generally thought to be a marker of memory T cells, which following exposure to antigen, provide fast and more severe immune responses. The primary analysis of tumor tissues of BC showed that besides being the most frequent, CD45RO+ lymphocytes were significantly higher in the higher-grade tissues. This increase was also observed in patients with stage II compared to stage I and muscle invasion. Given the protective role of memory cells in antitumor responses and their association with better prognosis in most cancers [13], the increase in the frequency of CD45RO+ cells can be basically interpreted by the continuous and prolonged contact of immune cells with tumor antigens and their efforts to remove cancer cells. However, Horn et al. have found no significant relationship between infiltrated subsets, including CD45RO+ cells, and BC clinicopathological features [10], but a similar association was obtained in our previous study on patients with breast cancer [9]. It seems that high-grade tumors with fast growth and less differentiation, represent more antigenic and phenotypic differences than the normal tissues, leading to more detection and recruitment of immune cells into tumors. In this regard, studies have shown that bladder tumors are highly immunogenic [14], and accordingly they can strongly induce immune responses at least at the early stages before tumor-specific suppression. Although CD3+ T cells are the main immune subset expressing CD45RO, it is important to note that CD45RO might be also found on a variety of other immune cells with different functional properties. Thereby, the evaluation of other markers and functional studies seems to be necessary to clarify the precise role of CD45RO+ cells in the context of tumors.

The presence of CD3^+^ T cells, as the main arm of cellular immunity and their association with desirable clinical outcomes, has been investigated in many cancers [15] including BC [10]. Our primary evaluation of CD3^+^ lymphocytes in tumor tissue from patients with BC showed that these cells, at the same range of CD45RO^+^, accounted for the highest level of investigated immune cells in the stromal and intratumoral regions of bladder tumors. The frequency of CD3^+^ lymphocytes was significantly higher in the intratumoral region of early-progressed tumors (T2 vs. T1 and stage II vs. I) and tumors having invasion to the muscular layer. Similar to CD45RO + cells, this increase could be a sign of stimulating the protective immune system following an inflammatory response induced by tumor cells in the first stages. However, studies on BC and other cancer types have shown that the growth and spread of metastatic tumors were associated with a decrease in the density of T cells [16, 17]. On the other hand, Karpina et al. have shown that the number of CD3^+^ lymphocytes at the time of tumor resection is significantly higher in patients who experience cancer recurrence [18]. Besides tumor type, this difference might be attributed to the stage of tumor progression. It is assumed that in the early stages, tumor growth is followed by immune system attempts to eradicate the tumor cells. But, following tumor progression and escape, the frequency of these cells is severely reduced or their function is suppressed through expression of inhibitory immune checkpoint molecules (i.e. PD-1/PD-Ls and Tim3) as we observed in our previous [19] and recent studies (unpublished data). It should be also noted that T lymphocytes are heterogeneous populations with very different effector functions, both protumorigenic [FOXP + regulatory T (Treg) and type 2 helper T (Th2)] and antitumorigenic (CD8^+^ cytotoxic T and Th1). Consistently, we observed that the frequency of protumorigenic subtypes, Th2 and FOXP3^+^ Treg cells significantly increased in the draining lymph nodes of patients with different cancers including BC [20, 21]. On the other hand, GM-CSF producing effector subset decreased in tumor-infiltrated lymph nodes [22]. Accordingly, in the present study, along with CD3^+^ T cells two other important functional markers of T cells, CD8 (cytotoxic marker) and FOXP3 (inhibitory molecule), were also investigated.

CD8^+^ cells, known as cytotoxic cells, are of the most important subsets of T cells in antitumor immune responses. They contain cytotoxic enzymes and induce death in tumor cells. Although their association with increased survival and recurrence prevention has been reported in many types of cancers, in urological malignancies, there are limited studies with controversial results. We found that, similar to other subtypes, the frequency of CD8^+^ lymphocytes in both areas of the tumor significantly increased with tumor growth from Ta/T1 to T2/T3 or from stage I to II. Masson-Lecomte et al. also have observed similar results in T1 versus Ta tumors [23]. Despite provoking immune response or increase in the recruiting factors, it is also possible that the tumor inhibitory microenvironment suppressed or changed the functionality of CD8^+^ T cells in a way to help tumor growth. Consistently, in our previous study on BC, we found that the frequency of protumorgenic subtypes of CD8^+^ T cells producing IL-4 and IL-17, remarkably increased in the tumor-draining lymph nodes along with tumor progression [24]. We also observed similar results in breast and salivary gland tumors [20, 25]. In the same way, Karpina et al. have shown that the number of CD8^+^ lymphocytes was higher in the early-stage patients who underwent transluminal resection and experienced recurrence [18]. Furthermore, Zhang et al. have indicated that considering the clinical stage, although CD8^+^ lymphocytes represented a favorable prognostic factor in non-organ-confined disease, they were an independent unfavorable factor in organ-confined ones [26]. It shows that the prognostic role of these cells and probably their function change during disease progression.

Along with effector T cells, some subpopulations exert inhibitory functions to regulate the immune response. In most cases, these regulatory cells express the FOXP3 transcription factor [27, 28]. Although there are controversial reports, the frequency of FOXP3^+^ Treg cells in most cancers has been associated with a worse prognosis [29]. The result of our study also showed an increase in the frequency of stromal FOXP3^+^ cells with tumor progression from Ta to T2, however, their frequencies decreased in T3. Similarly, in our previous studies on bladder and breast tumors, we observed that the frequency of FOXP3^+^ Treg cells in the draining lymph nodes significantly increased in patients with tumor-affected nodes [20, 21]. Controversial observations have been also reported in urothelial carcinoma but there are some reports which consistently showed a poor prognosis in patients with severe infiltration of Treg cells in their tumor tissues [8, 30]. In addition, Murai et al. have found that in the non-metastatic tissues, specimens with the high percentage of FOXP3^+^CD3^+^ cells had higher recurrence, suggesting a role for the contribution of these cells in tumor progression [30].

We next compared the frequencies of immune cells in tumor tissues between alive and dead patients. Frequency analyses did not represent any significant differences in both groups. None of the investigated markers showed a direct association with survival time, as well. Meanwhile, Horn et al. have found a correlation between lower OS and higher ratios of FOXP3^+^/CD3^+^ and FOXP3^+^/CD8^+^, but higher levels of CD3^+^ and CD8^+^ showed a greater tendency for better survival [10]. Sharma et al. have also found that patients with higher infiltrated CD8^+^ cells had better free-disease and overall survival, indicating a predictive role for CD8^+^ lymphocytes in BC [31], however, in our study, such associations were not observed. In a study by Winerdal et al., FOXP3 expression in tumor cells was associated with decreased survival, though its expression in tumor‐infiltrating lymphocytes correlated with a positive prognosis [8]. They first considered the FOXP3 as an activation marker for T cells rather than regulatory molecule, but the results of their recent study confirmed that these cells were committed Tregs and inhibited the metalloproteinase 2, a pro-invasive factor in the tumor microenvironment, which could explain observed positive correlation with patients’ prolonged survival [32]. However, in none of these studies, the tumor areas were separately investigated, while Yu et al. have observed that in patients with advanced BC, those with the higher density of CD3^+^ and CD8^+^ in the invasive margins showed better OS and higher DSF in case of more density of CD8^+^ [11]. This observation was consistent with the previous studies on BC which have found CD8^+^ as a favorable prognostic factor [16, 33, 34].

## Conclusion

We collectively observed a high degree of variation in the prevalence of immune cells in both stromal and intratumoral areas of bladder tumors. Statistical analysis showed that frequency of immune cells especially CD45RO+ , CD3+ and CD8+ lymphocytes were significantly higher in early-progressed tumors. This increase can be basically interpreted by the continuous and prolonged contact of immune cells with tumor antigens, their activation, increase in the recruiting factors and their efforts to eliminate tumor cells. However, with tumor progression to the late stages, tumor inhibitory microenvironment suppress or change the functionality of effector and memory immune cells in a way to help tumor growth. In addition, studies confirmed that the heterogeneity in the tumor microenvironment even in two patients with the same type of cancer, the tumor microstructure, distribution of immune cells in the margin and center of the tumor, secondary lymphoid structures, as well as the type of inflammatory cells and their functional status, have a great impact on the prognosis. Since low sample size in our study might reduce the power of the study, we suggest more functional studies with larger sample sizes to reveal the real status of the immune system in patients with bladder cancer.

## Data Availability

The datasets used and/or analyzed during the current study available from the corresponding author on reasonable request.
